# Item and domain network structures of the Resilience Scale for Adults in 675 university students

**DOI:** 10.1017/S2045796019000222

**Published:** 2019-04-22

**Authors:** G. Briganti, P. Linkowski

**Affiliations:** 1 Department of Clinical Epidemiology and Biostatistics, School of Public Health, Université libre de Bruxelles, Route de Lennik 808, 1070 Brussels, Belgium; 2 Department of Pathophysiology, École Supérieure de la Santé, Place du Chateau 3, 1014 Lausanne, Switzerland

**Keywords:** Behaviour, community detection algorithms, psychometrics

## Abstract

**Aims:**

The Resilience Scale for Adults (RSA) is a questionnaire that measures protective factors of mental health. The aim of this paper is to perform a network analysis of the RSA in a dataset composed of 675 French-speaking Belgian university students, to identify potential targets for intervention to improve protective factors in individuals.

**Methods:**

We estimated a network structure for the 33-item questionnaire and for the six domains of resilience: *perception of self*, *planned future*, *social competence*, *structured style*, *family cohesion* and *social competence*. Node predictability (shared variance with surrounding nodes in the network) was used to assess the connectivity of items. An exploratory graph analysis (EGA) was performed to detect communities in the network: the number of communities detected being different than the original number of factors proposed in the scale, we estimated a new network with the resulting structure and verified the validity of the new construct which was proposed. We provide the anonymised dataset and code in external online materials (10.17632/64db36w8kf.2) to ensure complete reproducibility of the results.

**Results:**

The network composed of items from the RSA is overall positively connected with strongest connections arising among items from the same domain. The domain network reports several connections, both positive and negative. The EGA reported the existence of four communities that we propose as an additional network structure. Node predictability estimates show that connectedness varies among the items and domains of the RSA.

**Conclusions:**

Network analysis is a useful tool to explore resilience and identify targets for clinical intervention. In this study, the four domains acting as components of the additional four-domain network structure may be potential targets to improve an individual's resilience. Further studies may endeavour to replicate our findings in different samples.

## Introduction

Resilience is understood as a positive adaptation despite significant adversities or trauma (Luthar, 2006). Resilience is a psychological construct which has been proven to be related to psychiatric disorders, such as anxiety, depression, substance abuse and obsessive-compulsive disorder (Hjemdal *et al*., 2011*a*, 2011*b*; Bonfiglio *et al*., 2016). In recent years, the construct of resilience has been conceived as an outcome rather than a trait, which highlights the ability to improve an individual's protective factors against mental illness (Chmitorz *et al*., 2018). In this framework, protective factors composing resilience compete with risk factors, for instance, adverse events (such as traumatic experiences, loss or neglect) which have been shown to be present in up to 50% of individuals under the age of 18 (Fritz *et al*., 2018). Other important factors influencing the framework of resilience involve age, social status and education (Aburn *et al*., 2016).

The Resilience Scale for Adults (RSA) is a psychometric questionnaire that assesses protective factors of mental health (Friborg *et al*., 2003). The RSA has been defined as one of the best resilience questionnaires with regard to psychometric ratings (Windle *et al*., 2011). Largely validated in Norwegian samples, the construct has undergone in the last decade cross-cultural validation in different countries, such as Belgium (Hjemdal *et al*., 2011*a*, 2011*b*), Iran (Jowkar *et al*., 2010), Italy (Bonfiglio *et al*., 2016) and Peru (Morote *et al*., 2017). The RSA measures six domains of resilience: (1) *perception of self* represents the confidence in oneself, one's own capabilities, judgement and decision-making (e.g. item 17 ‘My judgement and decisions I trust completely’); (2) *planned future* identifies goal-oriented individuals (e.g. item 32 ‘My goals for the future are well thought through’); (3) *social competence* represents the ability to adapt in social environments (e.g. item 21 ‘Meeting new people is something I am good at’); (4) *structured style* identifies with organised individuals who follow routines (e.g. item 23 ‘When I start on new things/projects, I prefer to have a plan’); (5) *family cohesion* measures the loyalty, support, optimism, mutual understanding and appreciation among family members (e.g. item 3 ‘My family understanding of what is important in life is very similar’) and (6) *social resources* identifies the availability of social support from friends and family (e.g. item 6 ‘I can discuss personal issues with friends/family members’).

These six domains are commonly understood as being effects of the construct of resilience itself, since they are measurable indicators of the construct.

However, in recent years, network theory has emerged as a way of studying psychological constructs as interacting entities (Borsboom, 2017). Such entities are uncovered in real-world data using network models, usually composed of pairwise interactions of its elements, and the constructs emerge from these connections (Borsboom and Cramer, 2013). Interactions between elements composing a network are often statistically represented as regularised partial correlations (Epskamp and Fried, 2018).

Several mental disorders have been analysed using a network perspective, such as posttraumatic stress disorder (Fried *et al*., 2018; Phillips *et al*., 2018), depression (Mullarkey *et al*., 2018), schizophrenia (Galderisi *et al*., 2018) and obsessive-compulsive disorder (Ruzzano *et al*., 2015). Network analysis has also been applied to several psychological constructs, such as personality (Costantini *et al*., 2015), empathy (Briganti *et al*., 2018), attitudes (Dalege *et al*., 2017), intelligence (van der Maas *et al*., 2006) and self-worth (Briganti *et al*., 2019). Other studies used innovative methods, including networks to harmonise rating scales (Haroz *et al*., 2016; Gross *et al*., 2018; Purgato *et al*., 2018).

Learning the network structure of a given construct (such as resilience) or mental disorder (such as posttraumatic stress disorder) is particularly relevant in clinical practice since it highlights potential clinical target that may affect multiple symptoms or elements composing the network (Fried *et al*., 2018); for instance, intervening on the connection between two components of the network is likely to modify the clinical presentations of said components (such as symptoms). In the specific case of resilience, which is considered a protection against mental disorders, learning the network structure of resilience components may highlight potential targets to strengthen the overall mental health of a given individual. In recent years, several intervention methods to foster resilience have been studied worldwide, but their efficiency is variable because of limited comprehension of this relevant psychological construct (Chmitorz *et al*., 2018).

A network analysis of resilience factors has also been proposed in two samples of adolescent subjects with and without childhood adversities (Fritz *et al*., 2018) and showed that childhood adversities impact the degree of connectivity of resilience factors.

Network components are usually answers of an observed group to items of a questionnaire, such as the RSA. A current challenge in network models when dealing with self-report scales is the redundancy of several items of a given questionnaire in measuring the same aspect of a construct (Fonseca-Pedrero *et al*., 2018); while addressing the meaning of a given connection between two items, their interaction will represent shared variance (and not a pairwise relationship) if they tend to measure the same thing (Fried and Cramer, 2017). In the case of the RSA, this challenge goes beyond the notion of a single items of the questionnaire and may apply to entire domains of the RSA: for instance, questions from both *perception of self* and *planned future* refer to one's own dispositional attributes and internal source of resilience and were original part of the same factor, which was called *personal competence* (Friborg *et al*., 2003). The same line of reasoning applies to *family cohesion* and *social resources*, even though originally distinct factors, since they represent an external source of resilience – that is, the support that the individual feels both within and without the family nucleus: furthermore, several items from *social resources* include the concept of family support (e.g. item 6 ‘I can discuss personal issues with friends/*family*’). Exploratory graph analysis (EGA) has emerged as a highly effective and reliable tool in network analysis when addressing the issue of recovering the number of factors in datasets (confirmatory factor analysis (CFA); Golino and Epskamp, 2017). An optimum solution proposed in the literature is to first explore the basic dimensionality of an instrument with an EGA then authenticate the suggested structure by performing a confirmatory factor analysis (Golino and Demetriou, 2017).

We aim to extend the conceptual framework of network analysis to the construct of resilience such as represented by the RSA and address the challenge of domain redundancy using both network models and structural equation models.

First, we want to explore the connectivity of the RSA as a network composed of its items, then study the connections arising among resilience domains, such as performed in recent network papers (Briganti *et al*., 2019). Second, we want to apply community detection algorithms and the EGA to the item network, explore then verify the suggested structure with CFA and network analysis.

Third, we want to measure node predictability which is an absolute measure of interconnectedness (Haslbeck and Fried, 2017) of a node in a network. Statistically speaking, node predictability represents the shared variance of a network component with surrounding components. Although performed on university students, exploring a network structure that shows how domains of resilience interact may have meaningful clinical implications as it highlights potential target to improve the overall protective factors of a given individual; also, it may serve as basis for future replication studies designed to identify the network structure of RSA in other samples.

## Methods

### Participants

The analyses in this paper are performed on a dataset composed of 675 university students from the French-speaking region of Belgium. In total, 59% of the students were women and 41% were men; subjects were 17–25 years old (*M*  =  19 years, s.d.  =  1.5 years).

### Measurement

The RSA (Table 1) is composed of 33 items that measure resilience in six domains: *perception of self*, *planned future*, *social competence*, *structured style*, *family cohesion* and *social competence*.

Table 1 has been removed as full appropriate permission was not obtained. A correction notice can be found at https://doi.org/10.1017/S2045796020000323. Please refer to the original reference for the table: Friborg O, Martinussen M and Rosenvinge JH (2006) Likertbased vs. semantic differential-based scorings of positive psychological constructs: a psychometric comparison of two versions of a scale measuring resilience. Personality and Individual Differences 40, 873-884.

The items are shuffled in the questionnaire. Item scoring is semantic and differential-based (Friborg *et al*., 2006): for instance, when scoring item 13 ‘My family is characterised by’, a minimum score of 1 is represented by the answer ‘Disconnection’ and a score of 7 is represented by the answer ‘Healthy cohesion’. Reversed-score items are included in the scale.

The dataset is anonymised and provided with the full R-code can be found at: 10.17632/64db36w8kf.2 to ensure complete reproducibility of the analyses carried out in the paper. This study was approved by the Ethical Committee of the Erasme university hospital.

### Network analysis

We used software R (version 3.5.1, open source, available at https://www.r-project.org/). Packages and functions to carry out the analysis include qgraph (Epskamp *et al*., 2012), glasso (Friedman *et al*., 2014) for network estimation and visualisation, mgm (Haslbeck and Waldorp, 2016) for node predictability, EGA (Golino and Epskamp, 2017) and igraph (Csardi and Nepusz, 2006) for community detection and bootnet (Epskamp *et al*., 2017) for stability. Complete information about package versions used in this paper can be found at: 10.17632/64db36w8kf.2.

#### Item network

We calculated correlations for the 33 RSA items and used the resulting correlation matrix as an input to estimate a Gaussian graphical model (GGM), a regularised partial correlation network (Epskamp and Fried, 2018). Graphical lasso (least absolute shrinkage and selection operator) was used to regularise the parameters resulting from the GGM, therefore avoiding the estimation of spurious connections (non-existing connections that may be present due to noisy information).

In the item network, nodes represent resilience items from the RSA questionnaire. Each node is surrounded by a pie chart representing node predictability (further described in the section ‘Network inference’).

Connections between nodes are called edges. An edge in a network is interpreted as the existence of an association between two nodes, controlling for all other nodes in the network. An edge between two items of the RSA may be statistically interpreted as following: if two given nodes X and Y share an edge XY in the network, and the observed group of subjects scores high on X, then the observed group is also more likely to score high on Y (Briganti *et al*., 2018). Each edge in the network represents either positive regularised partial correlations (visualised as blue edges) or negative regularised partial correlations (visualised as red edges). The thickness and colour saturation of an edge denotes its weight (the strength of the association between two nodes).

The Fruchterman–Reingold algorithm places the items in the network based on the sum of connections of a given node with other nodes (Fruchterman and Reingold, 1991).

#### Six-domain network

To assess the overall connectedness of the domains of resilience as described in the RSA (Hjemdal *et al*., 2011a, 2011b), we used the methodology described in recent papers (Briganti *et al*., 2019) and estimated a factor model using CFA for each of the six RSA domains. We then used the factor scores obtained to estimate an additional GGM.

#### Network stability

Network stability is composed of several state-of-the-art analyses which are necessary to safely interpret results from a network analysis. We estimated 95% confidence intervals (CI) of the edge weight through bootstrapping (Epskamp *et al*., 2017; 2000 bootstraps were used) and performed an edge weight difference test to answer the question ‘is edge A significantly stronger than edge B’.

#### Network inference

We estimated node predictability for the 33 RSA items and for the six domains. Node predictability (Haslbeck and Fried, 2017) represents shared variance of a given node with surrounding nodes in a network. Node predictability is an absolute measure of the interconnectedness of network nodes (Fried *et al*., 2018). Other measures of inference frequently used in the network literature such as strength centrality (Boccaletti *et al*., 2006) or expected influence (Robinaugh *et al*., 2016) can only address the relative importance of nodes (Briganti *et al*., 2019) and are therefore less informative when it comes to address the issue of interconnectedness; that is why we decided not to use these measures in this paper.

One interpretation of node predictability that has been previously described in the literature (Briganti *et al*., 2019) is that of the upper bound of controllability: this measure can provide an estimate of how much a node X can be influenced by all other nodes if we assume that all edges that node X shares with other nodes are directed towards X.

To explore the dimensionality of the RSA in our sample we performed an EGA on the item network. EGA uses the walktrap algorithm to detect communities. This algorithm is based on the principle that adjacent nodes tend to belong to the same community (Yang *et al*., 2016), was shown to have high accuracy in simulation studies (Golino and Epskamp, 2017) and used in empirical network papers (Briganti *et al*., 2018).

#### Four-domain network

Because we detected a different structure – composed of four domains instead of six as the one originally proposed (Hjemdal *et al*., 2011*a*, 2011*b*), we used CFA to estimate a four-factor model and used the resulting factor scores to estimate a four-domain network; because the network estimation procedure selected the network (out of a 100 networks) with the lowest tuning parameter (called *λ* value), we lowered the *λ* value to follow standard recommendations. We also calculated node predictability for the four nodes of the resulting network.

## Results

### Item network

**Fig. 1.**
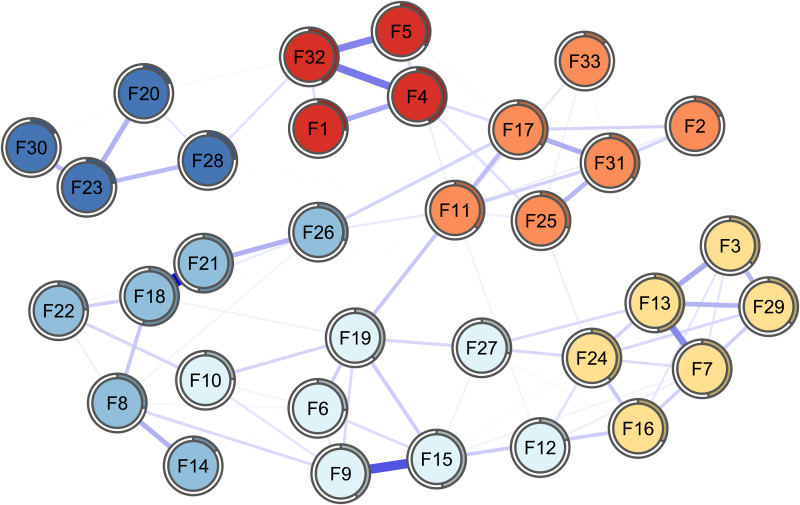

Figure 1 represents the item network: to render the visualisation more readable, we hid all edges smaller than 0.05 (one-tenth of the value of the maximum edge weight). Overall, the 33 items from the RSA form a network of positively connected nodes. The strongest connection (0.5) is the edge between node 18 (‘New friendships are something I make easily’) and node 21 (‘Meeting new people is something I am good at’), both belonging to *social competence*. Two other examples of strong connections are edge 9–15 belonging to *social resources* (‘Those who are good at encouraging me are some close friends/family’; ‘I get support from friends/family members’) and edge 4–32 belonging to *planned future* (‘I feel that my future looks very promising’; ‘My goals for the future are very thought through’). These examples of highly connected nodes reflect the challenge of items representing the same aspect of a construct and are discussed in the section ‘Discussion’.

However, several edges connect different domains of resilience. For instance, edge 11–19 connects *perception of self* and *social resources* (‘My personal problems I know how to solve’; ‘When needed, I have always someone who can help me’) and edge 17–26 connects *perception of self* and *social competence* (‘My judgement and decisions I trust completely’; ‘For me, thinking of good topics of conversation is easy’).

### Six-domain network

**Fig. 2.**
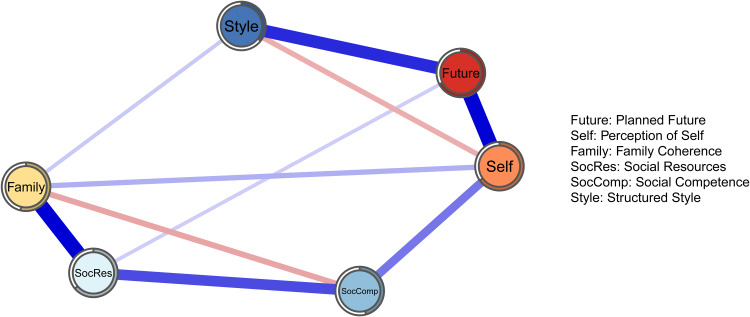

Figure 2 illustrates the six-domain network of resilience. This network reports considerably stronger connections because of disattenuation due to measurement unreliability. This issue is to be expected when dealing with a GGM based on correlations between factor scores and has been previously described in the literature (Briganti *et al*., 2019).

The strongest connections are found between *social resources* and *family cohesion* (0.59), and between *planned future* and *perception of self* (0.58). Two negative connections are found between *structured style* and *perception of self* (0.19) and between *family cohesion* and *social competence* (0.21). Several domains present no direct connection with each other, such as *structured style* and *social competence* or *social resources* and *perception of self*; from a network perspective, that means that the two domains are conditionally independent from each other.

We performed a CFA to assess the validity of the six-domain structure. Root mean square error of approximation (RMSEA) is 0.047 (cut-off for good fit <0.06) and the Standardised root mean square residual (SRMSR) is 0.058 (cut-off for good fit <0.08); Cronbach's *α* is 0.64 (>0.8 for good fit); comparative fit index (CFI) is 0.87 (>0.9 for good fit) and the *p*-value for the *χ*^2^ fit test is 0 (>0.05 for good fit; Schreiber, 2017). The full analyses can be found at: 10.17632/64db36w8kf.2.

### Network stability

State-of-the-art analyses carried out on all network models estimated in this paper can be found at: 10.17632/64db36w8kf.2. Bootstrapped 95% edge weight CI shows that the edge weights are accurately estimated, and the edge weight difference tests report that stronger edges can be safely interpreted as to be stronger that other edges in both the item network and the six-domain network, but do not statistically differ from each other in the six-domain network. For instance, one cannot safely interpret the edge between *social resources* and *family cohesion* to be stronger than the edge between *perception of self* and *planned future*.

### Network inference

#### Node predictability

Node predictability was estimated in both the item network and the six-domain network. In the item network, the two nodes with the highest node predictability are node 18 (‘New friendships are something I make easily’; 0.54) and 21 (‘Meeting new people is something I am good at’; 0.53), which both belong to *social competence* and also share the strongest edge in the network. The node with the lowest node predictability is node 33 which belongs to *perception of self* (‘Events in my life that I cannot influence I manage to come to terms with’; 0.13). Mean node predictability is 0.32, which means that on average, items from the RSA share 32% of their variance with surrounding nodes.

In the six-domain network, *planned future* shows the highest node predictability (0.67) and *structured style* is the least predictable node (0.36). The mean node predictability is 0.55, which means that on average, domains present 55% of shared variance.

#### Community detection

**Fig. 3.**
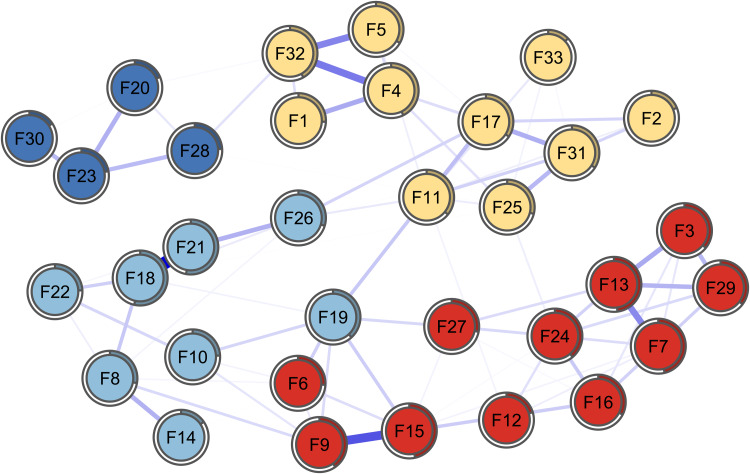

The EGA and walktrap algorithm applied to the item network report four communities of items instead of six as proposed in the original scale. The membership assigned to each item is displayed in Table 1 (column ‘WC’). To visualise the differences between communities, we first reestimates the network with a different colour palette, each colour indicating a community of items as detected by the algorithm as shown in Fig. 3.

Overall, items from *perception of self* and *planned future* form a new community, that we identify as *personal competence*, referring to one of the first versions of the RSA (Friborg *et al*., 2003); the same process applies to items from *social resources* and *family cohesion*, forming a new community that we identify as *support* since it is an aspect of resilience that the two domains represent.

Items 10 (‘The bonds among my friends are strong’) and 19 (‘When needed, I have always someone who can help me’) switch communities, going from *social resources* to *social competence*.

### Four-domain network

As suggested in the literature (Golino and Demetriou, 2017), we performed a CFA to assess the validity of the proposed structure. RMSEA is 0.064 (cut-off for good fit <0.06) and the SRMSR is 0.074 (cut-off for good fit <0.08); Cronbach's *α* is 0.64 (>0.8 for good fit); CFI is 0.74 (>0.9 for good fit) and the *p*-value for the *χ*^2^ fit test is 0 (>0.05 for good fit). The full analyses for the four-domain network can also be found at: 10.17632/64db36w8kf.2.

**Fig. 4.**
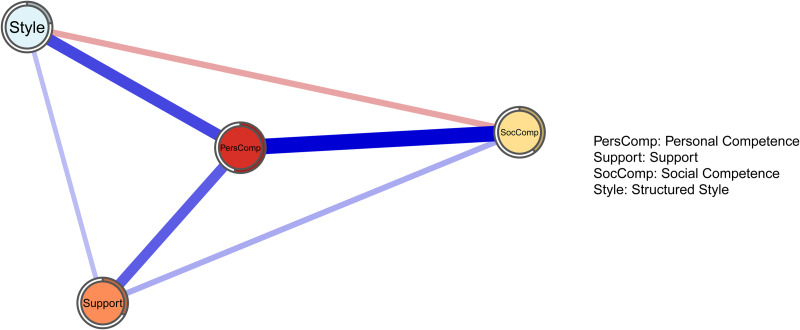

Figure 4 represents the four-domain network. Stability analyses carried out in this network show that stronger edges are significantly stronger than other edges. *Personal competence* is the most interconnected node, which is represented with the strongest positive connections with the three other domains (0.5 with *social competence*, 0.37 with *structured style* and 0.32 with support), and with a node predictability of 0.54. The node with the lowest node predictability is *structured style* (0.22). The mean node predictability for the four-domain network is 0.37. A negative edge is found between *structured style* and *social competence*.

## Discussion

This paper is, to our knowledge, the first work to report a network analysis of the psychological construct of resilience as conceived in RSA. The different analyses carried out bring new and interesting information on the construct, reporting overall that resilience is formed of interacting components which are not mere consequences of a latent variable. If the network structures presented in this work were to replicate in different samples, interventions to improve protective factors in individuals may become more efficient by acting on meaningful targets, such as two highly connected nodes in the resilience network.

The item network shows that the strongest edges are shared between items representing overall the same aspect of a domain: such connections must therefore be interpreted as shared variance between items, such as reported in recent papers that further address the issue (Fried and Cramer, 2017) and propose solutions such as estimating a network of domains instead of a network of items (Briganti *et al*., 2019). However, in the case of the RSA, the item network sheds light on the connectivity between items from different subscales: items from the RSA in our sample therefore form a complex system of mutual interactions that actively contribute to the construct of resilience itself. From a network perspective, this means the observed group is likely to similarly answer items that present a connection in the resilience network, after controlling for all other items in the network (Briganti *et al*., 2018). Items from the RSA also have different levels of importance in the network; this information is provided by node predictability, which is an absolute measure of the interconnectedness of a node (Haslbeck and Fried, 2017). In the item network, two nodes from the *social competence* domain (18 and 21) show the highest predictability, sharing over 50% of variance with surrounding nodes in the network structure: however, as addressed in the section ‘Results’, the two nodes with the highest node predictability are also the nodes sharing the strongest edge in the network; the high predictability is therefore largely influenced by the presence of one very strong edge, which is also a known feature influencing centrality criteria.

The six-domain network further helps us explore the connectivity and importance of the protective factors as described in the most recent version of the RSA. Domains of the RSA form a heterogeneous system with positive and negative connections: this further supports the theory that domains of resilience are not interchangeable measures of resilience; the construct arises from the connections among domains. For instance, two negative connections exist, the first between *structured style* and *perception of self*, and the second between *family cohesion* and *social competence*. Negative edges are a rare finding when dealing with a network approach of psychological constructs; a recent paper (Briganti *et al*., 2019) addressed the issue of interpreting negative edges in the case of a domain network such as the six-domain network of RSA estimated in this paper. From a theoretical point of view, we may interpret the negative edge between social competence and family cohesion as follows: knowing that an individual's resilience is strongly drawn from the ability to rapidly adapt in different social context, that individual's resilience is less likely to be drawn from the support originating from a cohesive family (and vice versa). The same line of reasoning applies to the negative connection between *structured style* and *perception of self*: knowing that an individual's resilience is drawn from routines and structure, his/her resilience is less likely drawn from confidence in own capabilities/decisions (and vice versa).

In the six-domain network, the strongest connections are found between *social resources* and *family cohesion*, and between *planned future* and *perception of self*. As mentioned in section the ‘Introduction’, these two couples of domains theoretically overlap, with several items measuring the same source of resilience; it is therefore not surprising that these domains are highly connected in a network structure. Domains of resilience predict each other well, with mean node predictability indicating 55% of shared variance on average. Planned future is the most important node in the resilience network according to the node predictability estimates (it has 67% of shared variance with surrounding nodes).

The EGA reported the existence of four communities in the item network, with a first new community, *personal competence*, emerging from *perception of self* and *planned future*, and a second new community, *support*, emerging from *social resources* and *family cohesion*. *Personal competence* (adding up items from *perception of self* and *planned future*) is, from a psychometric point of view, not a surprising finding: the two communities composing the new domain were originally a single factor (Friborg *et al*., 2003). However, *social resources* and *family cohesion* were originally proposed as distinct factors since the first published version of the scale, which makes this analysis an interesting finding.

In the four-domain network, the new *personal competence* community is the most interconnected node, sharing the three strongest connections in the network and 54% of its variance with the three surrounding domains. A negative edge is found between *structured* style and *social competence*, two domains unconnected in the six-domain network: from a theoretical point of view, it is plausible to consider that in people whose resilience depends on a structured life based on routines, being able to adapt in social situations is a less important source of resilience, controlling for all other sources (and vice versa). On average, nodes in the four-domain network are less predictable then domains in the six-domain network, with 37% of shared variance.

However, when comparing results from the CFA of both the six-factor model and the four-factor model (such as suggested by Golino and Demetriou, 2017), the six-factor model presents with better indicators than the four-factor model. This being the first network approach to this particular scale of resilience, future papers may endeavour to replicate these findings in other samples while comparing the original six-factor structure with structures proposed from EGA.

Our analyses should be interpreted in light of several limitations. First, our dataset is composed of university students, which may likely limit the generalisation of our findings to different samples. Second, because this is a cross-sectional study, we cannot infer whether a given node (item or domain) causes or is caused by another node to which it is connected; determination of causality requires time-series which may be interesting in follow-up studies of, for instance, young individuals with and without childhood adversities (Fritz *et al*., 2018).

## Conclusions

Network analysis is a useful tool to explore psychological constructs such as resilience as represented in questionnaires such as the RSA. Future studies in resilience may also endeavour to study the differences between network structures and node predictability in healthy subjects and in individuals with psychopathology. If the same network structures presented in this study were to replicate in different samples, target for clinical interventions such as highly connected nodes in the domain structures (for instance, the nodes connecting in the four-domain network structure) may be identified.

## Availability of Data and Materials

Data and materials for this study (data, code) are available via this link: 10.17632/64db36w8kf.2.
