# Item and domain network structures of the Resilience Scale for Adults in 675 university students – CORRIGENDUM

**DOI:** 10.1017/S2045796020000323

**Published:** 2020-04-06

**Authors:** G. Briganti, P. Linkowski

**Affiliations:** 1 Department of Clinical Epidemiology and Biostatistics, School of Public Health, Université libre de Bruxelles, Route de Lennik 808, 1070 Brussels, Belgium; 2 Department of Pathophysiology,École Supérieure de la Santé, Place du Chateau 3, 1014 Lausanne, Switzerland

Table 1 has been removed from the aforementioned article as full appropriate permission was not obtained. Please refer to the original reference for the table: Friborg O, Martinussen M and Rosenvinge JH (2006) Likert-based vs. semantic differential-based scorings of positive psychological constructs: a psychometric comparison of two versions of a scale measuring resilience. Personality and Individual Differences 40, 873–884.

The authors apologise for this error.
